# The Start2Bike program is effective in increasing health-enhancing physical activity: a controlled study

**DOI:** 10.1186/s12889-017-4523-1

**Published:** 2017-06-29

**Authors:** Linda Ooms, Cindy Veenhof, Dinny H. de Bakker

**Affiliations:** 1 Netherlands Institute for Health Services Research (NIVEL), PO Box 1568, 3500 BN, Utrecht, The Netherlands; 2 Physical Therapy Research, Program in Clinical Health Sciences & Department of Rehabilitation, Nursing Science and Sport, Brain Center Rudolf Magnus, University Medical Center Utrecht, PO Box 85500, 3508 GA, Utrecht, The Netherlands; 3 Utrecht University of Applied Sciences, Faculty of Health Care, PO Box 12011, 3501 AA, Utrecht, The Netherlands; 4 Scientific Center for Transformation in Care and Welfare (Tranzo), Tilburg University, PO Box 90153, 5000 LE, Tilburg, The Netherlands

**Keywords:** Sporting program, Organized sports, Sports club, Mountain biking, Road cycling, Health-enhancing physical activity, Controlled study, Follow-up

## Abstract

**Background:**

The sports club is seen as a new relevant setting to promote health-enhancing physical activity (HEPA) among inactive population groups. Little is known about the effectiveness of strategies and activities implemented in the sports club setting on increasing HEPA levels. This study investigated the effects of Start2Bike, a six-week training program for inactive adults and adult novice cyclers, on HEPA levels of participants in the Netherlands.

**Methods:**

To measure physical activity, the *S*hort *QU*estionnaire to *AS*sess *H*ealth-enhancing physical activity was used (SQUASH). Start2Bike participants were measured at baseline, six weeks and six months. A matched control group was measured at baseline and six months. The main outcome measure was whether participants met the Dutch Norm for Health-enhancing Physical Activity (DNHPA: 30 min of moderate-intensity activity on five days a week); Fit-norm (20 min of vigorous-intensity activity on three days a week); and Combi-norm (meeting the DNHPA and/or Fit-norm). Other outcome measures included: total minutes of physical activity per week; and minutes of physical activity per week per domain and intensity category. Statistical analyses consisted of McNemar tests and paired t-tests (within-group changes); and multiple logistic and linear regression analyses (between-group changes).

**Results:**

In the Start2Bike group, compliance with Dutch physical activity norms increased significantly, both after six weeks and six months. Control group members did not alter their physical activity behavior. Between-group analyses showed that participants in the Start2Bike group were more likely to meet the Fit-norm at the six-month measurement compared to the control group (odds ratio = 2.5; 95% confidence interval (CI) = 1.1–5.8, *p* = 0.03). This was due to the Start2Bike participants spending on average 193 min/week more in vigorous-intensity activities (b = 193; 95% CI = 94–293, *p* < 0.001) and 130 min/week more in sports activities (b = 130; 95% CI = 82–178, *p* < 0.001) than control group members.

**Conclusions:**

Start2Bike positively influences HEPA levels of participants by increasing participation in sport. A relatively short sporting program, offered by a sports club, can be used to encourage less active people to engage in and continue sport at HEPA levels. Overall, sport can contribute to health through increased HEPA and the sports club can serve as a setting to stimulate this.

**Electronic supplementary material:**

The online version of this article (doi:10.1186/s12889-017-4523-1) contains supplementary material, which is available to authorized users.

## Background

Participation in regular physical activity can bring a wide range of health benefits that impact upon the population. These benefits go beyond physical health to include other benefits, such as improved cognitive function, quality of life, personal wellbeing and social functioning [1–7]. To receive these health benefits, adults should undertake a minimum of 30 min of moderate-intensity physical activity on five days per week or 20 min of vigorous-intensity physical activity on three days per week. Also, an equivalent combination of both moderate- and vigorous-intensity physical activity is possible [5, 8]. However, research suggests that 31% of adults worldwide and 34% of Dutch adults do not meet these levels of health-enhancing physical activity (HEPA) [7, 9]. These people are at higher risk of developing chronic diseases and premature death [1–7].

Participation in sports activities at a sports club can contribute considerably to HEPA levels of individuals [10]. In Europe, 12% of the population is a sports club member. This percentage is even higher in the Netherlands (27%) [11]. Due to their wide reach, social and informal educational nature, sports clubs have great potential to promote a healthy and active lifestyle in the population [12, 13]. Indeed, health professionals and policy makers see the sports club as a new relevant setting for programs and strategies to increase HEPA among inactive population groups [14–18].

More traditional institutional settings, like universities and workplaces, have already been used for health promotion. Settings-based health promotion is based on the idea that health behaviors of individuals are influenced by the places in which they live, work and play and the factors interacting in those places (i.e. environmental, organizational and personal factors). Important aims of this approach are among others: 1) creating supportive and healthy environments in order to make ‘the healthy choice, the easy choice’ and 2) integrating health promotion in the daily strategies and activities of the setting [19–22]. However, health promotion in general, and the promotion of HEPA among inactive groups in particular, is still a relatively new concept for sports clubs. Their focus is mainly on providing training, competition and elite sports [14, 16].

Nonetheless, a few examples of HEPA promotion strategies and activities in sports clubs can be found in the literature. In Australia, for instance, they focused on the development of healthy (e.g. healthy eating, responsible serving of alcohol) and welcoming sports club environments as a means to increase sport participation by less active population groups. Also, the implementation of sporting programs that involved cross-sectoral partnerships (i.e. between sports clubs and other sectors, like health, education and recreation) was advocated [14–16]. In the Dutch context, National Sports Federations received funding within the National Action Plan for Sport and Exercise (NAPSE) to develop sporting programs adapted to the needs and abilities of inactive people [23]. These programs had to be integrated in the daily activities of their affiliated clubs. In this regard, the Netherlands Tour Cycling Union (NTFU) initiated Start2Bike, a six-week training program for inactive adults and adult novice cyclers. Participants are learned the basic skills of mountain biking or road cycling. Subsequently, they are encouraged to continue cycling in a beginner’s group at the club or as a member of the NTFU.

To date, research concerning HEPA strategies and activities in the sports club setting focused predominantly on implementation matters, like organizational readiness, partnership and capacity-building strategies and factors influencing implementation [14–16, 23]. However, still little is known about the effectiveness of these initiatives on increasing HEPA levels [24]. There is a request for an evaluation of activities in controlled studies, assessing both short- and longer-term effects [25, 26]. Until now, the only answer to this request was the evaluation of the Start to Run program, a six-week training program for novice runners, initiated by the Dutch Athletics Organization and implemented by local athletics clubs [27]. It proved to be effective in increasing HEPA levels of participants, with 69.0% of participants still engaged in running 4.5 months after they finished the program. However, it was stated that further research was needed to determine whether these results could be generalized to other sports and sporting programs. Therefore, this study aimed to investigate the short- and longer-term effects of the Start2Bike program on HEPA levels of participants in a controlled study design.

## Methods

### Study design

To determine the effects of Start2Bike on HEPA levels of participants, a controlled study design was used. For comparability purposes, this study used the same data collection and analyses methods as those applied in the Start to Run study [27]. Start2Bike participants subscribed for the program on a voluntary basis. Subsequently, they were asked to participate in this study. They were not subjected to procedures, nor were they obligated to follow certain behavioral rules. Therefore, consistent with Dutch legislation, medical ethics committee approval was not required [28]. This study was performed according to ethical guidelines (i.e. with regard to principles like informed consent, enabling participation, avoiding adverse consequences, avoiding undue intrusion, confidentiality and data protection) [29]. Privacy procedures were conform Dutch Data Protection Authority regulations. For reporting of results, the Transparent Reporting of Evaluations with Nonrandomized Designs (TREND) group reporting standards were used as a guidance [30].

### Study population

#### Start2Bike participants

Start2Bike is aimed at inactive adults and adult novice cyclers (i.e. mountain biking or road cycling). Dutch sportive cycling clubs offer the program twice a year (in spring and autumn) at 83 different locations. Recruitment of participants is done by the clubs in different ways, namely by the distribution of leaflets and posters, advertisements in local newspapers and word of mouth. This study included 260 adults (from the different Start2Bike locations) who had subscribed for the Start2Bike program in spring 2009, with email addresses provided by the NTFU. These persons received an email with study information and a link to an online baseline questionnaire. The Start2Bike participants provided consent for participation in this research by completing this questionnaire.

#### Control group

To control for possible changes in physical activity behavior in the Dutch adult population (i.e. physical activity changes caused by other factors than the Start2Bike program, like seasonal influences), members of the Dutch Health Care Consumer Panel served as control group. This panel consists of approximately 3000 adults (≥ 18 years) and forms a representative sample of the Dutch adult population. The panel is used to record views about and experiences with health care and other related topics [31]. In this study, initially 1328 panel members were included. Control group participants did not receive any intervention. Furthermore, it was questioned whether they had participated in the Start2Bike program or any of the other NAPSE sporting programs before or during the research period, because this could bias the results. Consequently, panel members who had done so were excluded from this study. In addition, the mean age and percentage of females was higher among questioned panel members compared with Start2Bike participants. Age and gender are known to influence physical activity levels [11]. Therefore, an age and gender matched control group was formed.

### Start2Bike program

The program was aimed at riding a mountain bike tour of 30 km or a road cycling tour of 70 km. In Table 1, a description of the training program can be found. The program lasted for six weeks. Each week consisted of a group session led by professional coaches and two individual cycling sessions, whereby rest days were scheduled after training days. For individual training sessions, it was advised to cycle at least with one other person. A group session (± 2 h) included an introductory part (± 30 min), core 1 (30 min), core 2 (45 min) and closure part (15 min). The introductory part consisted of a welcoming and explanation of the training, an equipment check (e.g. cycle, helmet) and warming-up (30 min). The warming-up was a combination of cycling, stretching and repeating of technical skills of the previous training. In core 1, a new technical skill was practiced and, in core 2, this was done with increasing cycling intensity and duration. Practice was ended with a cooling-down (10 min), which consisted of cycling at a slow speed and stretching. At the closure part, the training was discussed, the bicycle was cleaned (when possible) and the coach(es) provided instructions for the individual training sessions to the participants. Theory items, like the risks and (health) benefits of cycling, prevention of injuries and physiological outcomes of training, were discussed before or during practice items. During individual training sessions, participants had to practice previous learned technical skills, whereby cycling distance was gradually increased during the training period. At the sixth guided training session, participants could practice and test their cycling skills in a test tour, before participating in a real NTFU mountain biking (30 km) or road cycling tour (70 km) (one week thereafter). Participants trained in a group of maximum twelve people. When there were more than twelve participants, the group was split. There were at least two professional coaches per guided training session and one coach per group of twelve people. The NTFU provided trainer courses, especially for the Start2Bike program. At the end of the program, participants were encouraged by their coach(es) (both verbally and through email) to continue mountain biking or road cycling in a beginner’s group at the club through club membership. They were also informed about the option to continue cycling through an individual membership of the NTFU. Participants brought their own bicycle and equipment. In some locations, it was possible to lend materials.

**Table 1:** Content Start2Bike sporting program

Content guided training	T	Theory items	Technical skills	Homework
Introduction (± 30 min): ▪ Welcome and discussing program of the day. ▪ Check of equipment (helmet, bicycle, clothes). ▪ Warming-up (30 min): cycling, stretching and repeating the technical skill from the previous training. Core 1 (30 min): ▪ Practicing a new technical skill. Core 2 (45 min): ▪ Practicing technical skills while increasing intensity and duration of cycling. ▪ Cooling-down (10 min): cycling and stretching. Closure (15 min): ▪ Discussing the training of today. ▪ Coach prescribes homework for the upcoming week. ▪ When possible: cleaning of bicycle. Theory items are discussed before or during practice items.	1	▪ What is mountain biking/road cycling? ▪ Risks and (health) benefits of cycling. ▪ Tuning of bicycle and helmet. ▪ Equipment (clothes, saddle, helmet, shoes, sun glasses). ▪ Safety during cycling.	Braking, how to use your gears	Practicing previous learned skills: braking, using gears. Mountain biking: two times cycling 10 km. Road cycling: two times cycling 25–45 km.
	2	▪ Proper foods and drinks before, during and after training.	Balance	Practicing previous learned skills: braking, using gears, balance. Mountain biking: two times cycling 10 km. Road cycling: two times cycling 25–45 km.
	3	▪ Physiological processes during exercise.	Riding curves	Practicing previous learned skills: braking, using gears, balance, riding curves. Mountain biking: two times cycling 15 km. Road cycling: two times cycling 30–50 km.
	4	▪ Prevention of injuries.	Overcoming obstacles (mountain biking) or cycling in a group (road cycling)	Practicing previous learned skills: braking, using gears, balance, riding curves, overcoming obstacles/cycling in a group. Mountain biking: two times cycling 20 km. Road cycling: two times cycling 40–55 km.
	5	▪ Physiological outcomes of training (e.g. improving strength, endurance, physiological adaptations to training). ▪ Training with a heart rate monitor.	Climbing & descending	Practicing previous learned skills: braking, using gears, balance, riding curves, overcoming obstacles/cycling in a group, climbing and descending. Mountain biking: two times cycling 25 km. Road cycling: two times cycling 40–60 km.
	6	▪ Stimulating continuation of cycling (e.g. at a sports club).	Test mountain biking or road cycling tour	Practicing all previous learned skills. Mountain biking: cycling 25 km and the last training 15 km. Road cycling: cycling 60 km and the last training 25 km.

T = Training session

### Outcome measures

Demographic data (i.e. age and gender) were collected from all study participants. To measure physical activity, the *S*hort *QU*estionnaire to *AS*sess *H*ealth-enhancing physical activity (SQUASH) was used. This tool is considered to be sufficiently reliable and valid to measure physical activity levels in an adult population [32]. The applied SQUASH procedure has been described in more detail elsewhere [27]. In short, the SQUASH contains questions about five domains of physical activity: 1) commuting activities, 2) leisure-time activities, 3) sports activities, 4) household activities, and 5) activities at work and school. The amount of time participants spent in each of the domain-specific activities was measured for an average week in the past month, using three main queries: days per week, average time per day and intensity (light, moderate, vigorous). Whether participants met Dutch physical activity norms was the main outcome measure derived from the SQUASH (i.e. meeting HEPA levels). According to these norms, adults should undertake a minimum of 30 min of moderate-intensity physical activity on five days per week (Dutch Norm for Health-enhancing Physical Activity: DNHPA) or 20 min of vigorous-intensity physical activity on three days per week (Fit-norm) for health benefits. Someone who meets at least one of the two norms adheres to the so-called ‘Combi-norm’, the third norm used in the Netherlands (see also Table 2). Dutch physical activity norms are based on international physical activity guidelines [5, 9]. Secondary outcome measures calculated included: total minutes of physical activity per week; and minutes of physical activity per week per domain and intensity category.

**Table 2:** Dutch physical activity norms for adults

Norm	Description
Dutch Norm for Health-enhancing Physical Activity (DNHPA)	*Adults (18–54 years):* Thirty minutes or more of at least moderate-intensity aerobic (endurance) physical activity (≥ 4 MET) on at least five days each week. *Adults (55 years and older):* Thirty minutes or more of at least moderate-intensity aerobic (endurance) physical activity (≥ 3 MET) on at least five days each week. A moderate-intensity aerobic physical activity requires a moderate amount of effort and noticeably accelerates the heart rate, e.g. brisk walking, gardening.
Fit-norm	*Adults (18–54 years):* Twenty minutes or more of vigorous-intensity physical activity (≥ 6.5 MET) on at least three days each week. *Adults (55 years and older):* Twenty minutes or more of vigorous-intensity physical activity (≥ 5 MET) on at least three days each week. A vigorous-intensity physical activity requires a large amount of effort and causes rapid breathing and a substantial increase in heart rate, e.g. running, fast cycling.
Combi-norm	Meeting the DNHPA and/or Fit-norm. An adult is physically active enough to improve and maintain health when he or she meets at least one of the above mentioned norms (i.e. the DNHPA or Fit-norm).

*MET* Metabolic equivalent

Physical activity measurements of Start2Bike participants were performed at baseline (*t* = 0), six weeks (*t* = 6 weeks: i.e. directly after they finished the program) and six months after baseline (*t* = 6 months: i.e. 4.5 months after they finished the program) using an online questionnaire. To enhance response for comparisons with the control group, all Start2Bike participants who returned the baseline questionnaire were invited to fill in the questionnaire at six months, irrespective if they had returned the questionnaire at six weeks. Physical activity measurements of control group participants were performed simultaneously at baseline (*t* = 0) using a postal questionnaire and six months (*t* = 6 months) using a postal or an online questionnaire. When necessary, a reminder was sent after a week (online forms) or two weeks (postal forms).

### Sample size

The sample size in this study was based on finding a change in HEPA. Mountain biking and road cycling are vigorous-intensity physical activities. Therefore, the Dutch Fit-norm (see Table 2) was used as reference. A sample size of 74 participants per group was needed to find a 20% difference between the Start2Bike group and the control group at the six-month measurement, assuming an alpha of 0.05 (two-sided) and a power of 0.80. With 260 and 1328 participants included in the study for the Start2Bike group and control group, respectively, it was assumed that an adequate number of participants was approached.

### Statistical analysis

The software program Stata (version 10.1, Stata Corporation, College Station, Texas) was used to perform statistical analyses. The main features of each group were described using descriptive statistics. A chi-squared test and an independent t-test were used to test between-group differences with regard to gender and age, respectively. McNemar tests (dichotomous measures) and paired t-tests (continuous measures) were performed to examine within-group changes in physical activity. Multiple logistic (dichotomous measures) and linear (continuous measures) regression analyses were used to test between-group changes in physical activity. In the regression analyses, physical activity level at six months was the dependent variable and group (Start2Bike group vs. control group, with the control group serving as reference category) the independent variable. To adjust for baseline physical activity, this variable was also added to the regression model as an independent variable. For instance, to test changes in meeting the Fit-norm between groups, the following variables were added to the logistic regression analyses: meeting the Fitnorm at six months (yes/no, dependent variable); group (Start2Bike vs. control group, independent variable); meeting the Fitnorm at baseline (yes/no, independent variable). In addition, more robust regression procedures were performed to examine whether the results (continuous measures) were influenced by outliers: this included the use of robust standard errors (i.e. bootstrap and Huber-White robust estimates of the standard errors). These latter procedures did not alter results and conclusions of this study significantly. Therefore, these results are not described in this article. The significance level for all analyses was set at *p* < 0.05.

## Results

**Fig. 1:**
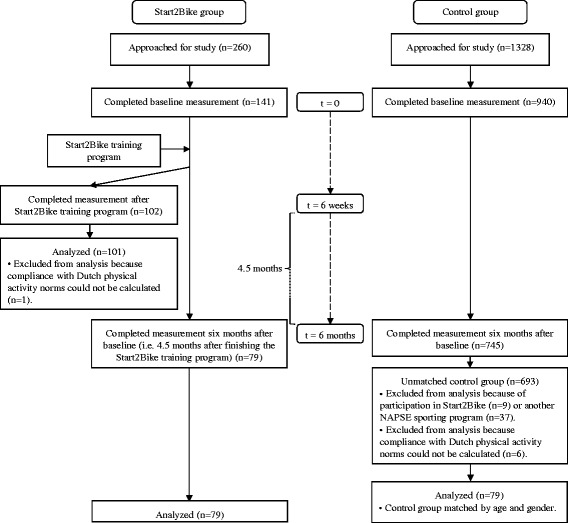
Participant flow through the study

Figure 1 presents a diagram of participant flow through the study. All three questionnaires were filled in by 72 Start2Bike participants (not presented in Fig. 1). However, to preserve study power and use data optimally, all available cases were used in the analyses, i.e. 101 (changes after six weeks) and 79 (changes after six months) Start2Bike participants, respectively. Non-response analyses showed that there were no baseline differences in demographic factors and physical activity behavior between Start2Bike participants who did and did not fill in the six-month questionnaire.

### Characteristics at baseline

Table 3 presents baseline characteristics of Start2Bike and control group participants. Two thirds of Start2Bike participants was male and the average age was 45 years (SD = 9). Gender and age characteristics, as well as baseline physical activity levels were comparable between the Start2Bike group and control group.

**Table 3:** Characteristics at baseline

	Start2Bike group^b^	Control group	*P*
Sample size (n)	79	79	
Age (years)			
Mean ± SD	45 ± 9	46 ± 9	0.55
Min-max	25–69	24–64	
Sex (%)			
Male	67.1	67.1	1.0
Female	32.9	32.9	
Dutch physical activity norms^a^, (%)			
Compliance with DNHPA	55.7	62.0	0.42
Compliance with Fit-norm	63.3	53.2	0.20
Compliance with Combi-norm	69.6	68.4	0.86
Physical activity by intensity, mean ± SD (min/week)			
Light-intensity activities	1940 ± 1313	1909 ± 1375	0.89
Moderate-intensity activities	329 ± 586	424 ± 613	0.32
Vigorous-intensity activities	358 ± 368	288 ± 420	0.27
Physical activity by domain, mean ± SD (min/week)			
Commuting activities	102 ± 226	130 ± 220	0.44
Leisure-time activities	346 ± 359	433 ± 495	0.22
Sports activities	173 ± 203	127 ± 248	0.21
Household activities	465 ± 769	732 ± 972	0.06
Activities at work and school	1581 ± 1038	1254 ± 1041	0.05
Total time spent in physical activity, mean ± SD (min/week)	2626 ± 1372	2622 ± 1490	0.99

*SD* Standard deviation

^a^Dutch physical activity norms:

- *DNHPA* Dutch Norm for Health-enhancing Physical Activity: Thirty minutes or more of at least moderate-intensity aerobic (endurance) physical activity on at least five days each week

- Fit-norm: Twenty minutes or more of vigorous-intensity physical activity on at least three days each week

- Combi-norm: Meeting the DNHPA and/or Fit-norm

^b^Start2Bike participants who completed the six-month measurement

### Physical activity changes

#### Physical activity changes after six weeks: Start2Bike participants

Physical activity levels of Start2Bike participants at the baseline and six-week measurement are shown in Table 4. Compliance with Dutch physical activity norms increased significantly (*p* < 0.001) from baseline to six weeks (DNHPA: 56.4% vs. 77.2%; Fit-norm: 66.3% vs. 86.1%; Combi-norm: 72.3% vs. 90.1%). Despite this increase, the total minutes per week of physical activity decreased (2678 ± 1322 min/week vs. 2137 ± 1037 min/week, *p* < 0.001). Furthermore, significant (*p* < 0.001) changes were observed within intensity categories: a decrease in light-intensity activities (2006 ± 1302 min/week vs. 1360 ± 912 min/week) and an increase in vigorous-intensity activities (359 ± 341 min/week vs. 532 ± 347 min/week). Within physical activity domains significant (*p* < 0.001) changes occurred for sports activities (increase: 189 ± 208 min/week vs. 337 ± 246 min/week) and activities at work and school (decrease: 1642 ± 981 min/week vs. 971 ± 651 min/week).

**Table 4:** Physical activity changes after six weeks: Start2Bike participants

Outcome measures	Start2Bike group (*n* = 101)		
	Baseline	After 6 weeks	*P* ^b^
Dutch physical activity norms^a^, (%)			
Compliance with DNHPA	56.4	77.2	<0.001*
Compliance with Fit-norm	66.3	86.1	<0.001*
Compliance with Combi-norm	72.3	90.1	<0.001*
Physical activity by intensity, mean ± SD (min/week)			
Light-intensity activities	2006 ± 1302	1360 ± 912	<0.001*
Moderate-intensity activities	314 ± 566	245 ± 393	0.10
Vigorous-intensity activities	359 ± 341	532 ± 347	<0.001*
Physical activity by domain, mean ± SD (min/week)			
Commuting activities	108 ± 210	102 ± 145	0.70
Leisure-time activities	311 ± 310	355 ± 341	0.15
Sports activities	189 ± 208	337 ± 246	<0.001*
Household activities	495 ± 788	426 ± 575	0.39
Activities at work and school	1642 ± 981	971 ± 651	<0.001*
Total time spent in physical activity, mean ± SD (min/week)	2678 ± 1322	2137 ± 1037	<0.001*

*SD* Standard deviation

^a^Dutch physical activity norms:

- *DNHPA* Dutch Norm for Health-enhancing Physical Activity: Thirty minutes or more of at least moderate-intensity aerobic (endurance) physical activity on at least five days each week

- Fit-norm: Twenty minutes or more of vigorous-intensity physical activity on at least three days each week

- Combi-norm: Meeting the DNHPA and/or Fit-norm

^b^ *P*-value for change in physical activity within the Start2Bike group

*Significant (*p* < 0.05) change in physical activity after six weeks within the Start2Bike group

#### Physical activity changes after six months: Within-group comparisons

Table 5 presents physical activity levels at the baseline and six-month measurement for both the Start2Bike group and control group. In the Start2Bike group, compliance with Dutch physical activity norms increased significantly from baseline to six months (DNHPA: 55.7% vs. 70.9%, *p* = 0.02; Fit-norm: 63.3% vs. 74.7%, *p* = 0.04; Combi-norm: 69.6% vs. 81.0%, *p* = 0.04). This was due to the Start2Bike participants spending significantly more minutes in vigorous-intensity activities (358 ± 368 min/week vs. 475 ± 405 min/week, *p* = 0.01) and sports activities (173 ± 203 min/week vs. 255 ± 208 min/week, *p* < 0.001). There were, however, no changes in the total minutes per week of physical activity (2626 ± 1372 min/week vs. 2535 ± 1372 min/week, *p* = 0.57). Physical activity levels of control group members did not change significantly within the six-month study period.

**Table 5:** Physical activity changes after six months: within-group comparisons

Outcome measures	Start2Bike group (*n* = 79)			Control group (*n* = 79)		
	Baseline	After six months	*P* ^b^	Baseline	After six months	*P* ^c^
Dutch physical activity norms^a^, (%)						
Compliance with DNHPA	55.7	70.9	0.02*	62.0	62.0	1.0
Compliance with Fit-norm	63.3	74.7	0.04*	53.2	55.7	0.79
Compliance with Combi-norm	69.6	81.0	0.04*	68.4	73.4	0.45
Physical activity by intensity, mean ± SD (min/week)						
Light-intensity activities	1940 ± 1313	1760 ± 1350	0.22	1909 ± 1375	1902 ± 1148	0.96
Moderate-intensity activities	329 ± 586	300 ± 507	0.56	424 ± 613	355 ± 558	0.32
Vigorous-intensity activities	358 ± 368	475 ± 405	0.01*	288 ± 420	255 ± 287	0.44
Physical activity by domain, mean ± SD (min/week)						
Commuting activities	102 ± 226	99 ± 142	0.87	130 ± 220	101 ± 182	0.24
Leisure-time activities	346 ± 359	335 ± 433	0.78	433 ± 495	359 ± 330	0.15
Sports activities	173 ± 203	255 ± 208	<0.001*	127 ± 248	105 ± 148	0.32
Household activities	465 ± 769	444 ± 659	0.70	732 ± 972	580 ± 805	0.10
Activities at work and school	1581 ± 1038	1449 ± 999	0.36	1254 ± 1041	1397 ± 995	0.27
Total time spent in physical activity, mean ± SD (min/week)	2626 ± 1372	2535 ± 1372	0.57	2622 ± 1490	2512 ± 1115	0.49

*SD* Standard deviation

^a^Dutch physical activity norms:

- *DNHPA* Dutch Norm for Health-enhancing Physical Activity: Thirty minutes or more of at least moderate-intensity aerobic (endurance) physical activity on at least five days each week

- Fit-norm: Twenty minutes or more of vigorous-intensity physical activity on at least three days each week

- Combi-norm: Meeting the DNHPA and/or Fit-norm

^b^ *P*-value for change in physical activity within the Start2Bike group

^c^ *P*-value for change in physical activity within the control group

*Significant (*p* < 0.05) change in physical activity after six months within the Start2Bike group

#### Physical activity changes after six months: Between-group comparisons

Table 6 presents the results of the between-group analyses. These analyses showed that participants in the Start2Bike group were more likely to meet the Fit-norm at the six-month measurement compared to the control group (odds ratio = 2.5; 95% confidence interval (CI) = 1.1–5.8, *p* = 0.03). This was due to the Start2Bike participants spending on average 193 min/week more in vigorous-intensity activities (b = 193; 95% CI = 94–293, *p* < 0.001) and 130 min/week more in sports activities (b = 130; 95% CI = 82–178, *p* < 0.001) than control group members. Both groups were comparable for the other physical activity measures.

**Table 6:** Physical activity changes after six months: between-group comparisons

Dichotomous outcome measures	OR (group variable)^b^	95% CI	*P* (group variable)
Dutch physical activity norms^a^			
Compliance with DNHPA	2.0	0.9–4.3	0.08
Compliance with Fit-norm	2.5	1.1–5.8	0.03*
Compliance with Combi-norm	1.7	0.7–4.0	0.23
Continuous outcome measures	b-coefficient (group variable)^b, c^	95% CI	*P* (group variable)
Physical activity by intensity			
Light-intensity activities	−157	−494-180	0.36
Moderate-intensity activities	−6	−145-132	0.93
Vigorous-intensity activities	193	94–293	<0.001*
Physical activity by domain			
Commuting activities	8	−37-54	0.72
Leisure-time activities	17	−87-121	0.75
Sports activities	130	82–178	<0.001*
Household activities	14	−160-189	0.87
Activities at work and school	−44	−351-263	0.78
Total time spent in physical activity	22	−329-373	0.90

*CI* Confidence interval, *OR* Odds ratio

^a^Dutch physical activity norms:

- *DNHPA* Dutch Norm for Health-enhancing Physical Activity: Thirty minutes or more of at least moderate-intensity aerobic (endurance) physical activity on at least five days each week

- Fit-norm: Twenty minutes or more of vigorous-intensity physical activity on at least three days each week

- Combi-norm: Meeting the DNHPA and/or Fit-norm

^b^To test between-group changes in physical activity, multiple (logistic or linear) regression analyses were performed. Physical activity level at six months was the dependent variable and group (the control group served as reference category) was the independent variable. Corrections were made for baseline physical activity levels

^c^Unstandardized regression coefficient

*Significant (*p* < 0.05) difference in physical activity between groups

## Discussion

### General findings

This study examined the effects of participation in the six-week Start2Bike program on HEPA levels of participants. Start2Bike participants increased HEPA levels, both at the six-week and six-month measurement. Physical activity levels of control group members did not change significantly within the six-month study period. Between-group analyses showed that the Start2Bike program resulted in higher HEPA levels of participants according to the Fit-norm. This was due to the Start2Bike participants spending more minutes per week in vigorous-intensity activities (intensity category) and sports activities (domain).

### Explanation of findings

Mountain biking and road cycling are vigorous-intensity sports activities. Therefore, the findings suggest that at least a part of the participants was still cycling 4.5 months after the last training session. In the six-month questionnaire, participants were also directly asked whether they were still cycling. Indeed, 75.9% of participants was still practicing the sport and 32.9% did this at a sportive cycling club [see Additional file 1 - Additional study results Start2Bike]. It should be noted, however, that these results do not reveal how much time was actually spent on cycling and it is possible that Start2Bike promoted participation in other sports activities as well. Nevertheless, participation in Start2Bike results in sport participation at HEPA levels and thus additional health benefits [5–8]. Moreover, considering the intensity of the practiced sports activities, there is evidence that high-intensity activities have more benefit for reducing cardiovascular disease and premature mortality than lower-intensity activities [33–35].

### Comparison with other studies

Mountain biking and road cycling are non-load bearing sports (i.e. less pressure is placed on joints and tendons as compared to, for example, running) which can be easily incorporated into daily routines (e.g. cycling from home to work). Consequently, they are also suitable activities for less active people. On the other hand, the sports require particular cycling skills and have the image of being fast, exciting and adventurous [36]. A ‘tough’ image of the sport can impede the recruitment of inactive people [23]. This may explain why these sports have not been widely used as physical activities in HEPA promotion strategies [e.g. 37]. Nevertheless, the positive health effects of cycling in general have been well documented in the literature [38, 39]. Therefore, there are a lot of cycling interventions aimed at promoting regular cycling (as opposed to sportive cycling), like town-wide media campaigns, cycle skills training and improvements in cycling infrastructure [40]. However, a recent systematic review about cycling interventions concluded that it is unclear whether these interventions result in an increase in physical activity [40].

On the other hand, the study findings are in support with the results of the evaluation of the Start to Run training program [27]. Furthermore, the study findings extent those results from running to sportive cycling. Consequently, it appears that a relatively short sporting program, implemented by sports clubs, can attract less active people, encourage them to participate in sport, and continue to practice sport at HEPA levels. However, there were also differences with regard to the participant population: The Start2Bike participants were more likely to be male (67.1% vs. 30.0%) and somewhat older (average age: 45 years vs. 40 years) compared with Start to Run participants. This may be inherent to the practiced sports. Nonetheless, this may have implications for the effectiveness of these sporting programs on increasing HEPA of different population subgroups. Therefore, in future research, it should be further studied which less active population subgroups (e.g. older adults vs. young adults, women vs. men, people with or without chronic disease) benefit most in terms of increasing HEPA levels. This will provide further insight into the usefulness of these sporting programs for particular subgroups.

### Practical implications and future directions

Sports clubs are seen as new relevant settings to increase HEPA among inactive population groups [14–18]. The discussed results show that sport can contribute to health through increased HEPA and the sports club can serve as a setting to stimulate this. However, for actual health benefits, continued participation in sport at HEPA levels is necessary. Therefore, also efforts should be placed to maintain participation. At the end of the programs, Start2Bike and Start to Run participants were personally encouraged by their coaches to continue practicing the sport in a beginner’s group at the sports club through club membership. At the six-month measurement, 32.9% of the Start2Bike participants was (still) member of the sports club who offered the Start2Bike program [see Additional file 1 - Additional study results Start2Bike]. For the Start to Run program, this percentage was somewhat higher (41.0%) [27]. Both running and sportive cycling are feasible sports that can be done anywhere and at any time. This may explain why a part of the participants was continuing the sport in non-organized forms. This can be different, however, for less feasible sports, like sports for which special facilities or equipment are needed (e.g. indoor (team) sports). Nevertheless, the sports club offers a social sporting context. Social support through interaction with other people at the club can be beneficial in maintaining sport behavior [12, 13, 41]. However, the actual amount of support that participants received as a club member was not measured. Therefore, it should be studied if and under what conditions participation in club sport can contribute to maintaining HEPA levels. Maintenance of behavior occurs when changes are sustained for a period of at least six months after cessation of an intervention [42]. Consequently, future research should also include multiple physical activity measurements over longer time periods to determine whether this sport behavior is continued.

### Strengths and limitations of the study

The Start2Bike program was developed by a sporting organization and implemented by local sports clubs, with participants voluntary participating. Consequently, this research reflects activities implemented in the real-world sports setting and research results are directly applicable to practice. These are strengths of this study. On the other hand, this non-intrusive study design precluded the use of more objective physical activity measures (e.g. accelerometers) and the analyses of independent program parts (e.g. individual sessions, group sessions, sport event) [27]. Furthermore, selection bias may have occurred because participation in the Start2Bike program was on a voluntary basis. It is possible that people who chose to participate were more motivated to increase HEPA than those who did not (choose to) participate. Therefore, the results may not be generalizable to less-motivated people, i.e. often the least-active ones. On the other hand, also adults who did not meet HEPA levels participated in the program, indicating that the program is appropriate for less active population groups. In addition, the voluntary character of participation is also a strength of the study. Behavior of participants was not forced and the Start2Bike study population was a sample of the actual Start2Bike population. This was confirmed by demographic data (i.e. gender and age) of the NTFU of the whole Start2Bike population in spring 2009 (*n* = 422). Consequently, the research was performed in a generalizable group. Not all participants contacted responded to all questionnaires. The percentage of participants dropping out of the study was, however, comparable to the drop-out of the Start to Run study [27]. In addition, non-response analyses showed that respondents did not differ from non-respondents with regard to baseline demographic factors and physical activity behavior. Thus, it is improbable that study results were influenced markedly by these losses to follow-up. Finally, although participation in vigorous-intensity sports is associated with many health benefits, some undesirable effects, like sports-related injuries, may also occur. Most cycling injuries can be prevented, however, by training well, ride safely and using protective gear (e.g. a helmet) [36]. Nonetheless, it is important to consider these possible adverse effects in future research.

## Conclusions

The study results show that the six-week Start2Bike program positively influences HEPA levels of participants by increasing participation in sport. In addition, the results support previous research that a relatively short sporting program, offered by sports clubs, can encourage less active people to engage in and continue sport at HEPA levels. Overall, sport can contribute to health through increased HEPA and the sports club can serve as a setting to stimulate this. Consequently, these results are of value to policymakers and sports practitioners who acknowledge the possibilities of sports clubs in health promotion. Future research should investigate whether sport behavior is maintained and if and under what conditions participation in club sport can support this. Also, the suitability of sporting programs for different less active population subgroups should be examined. In this way, policy makers and sports practitioners can make well-informed choices regarding the contribution of this setting to a healthy and active lifestyle.

## Additional file

Additional file 1: Additional study results Start2Bike. In this file, some additional results of the Start2Bike study (not shown in the results section of this article) are presented. (DOC 27 kb)

## Abbreviations

CI: Confidence interval
DNHPA: Dutch Norm for Health-enhancing Physical Activity
HEPA: Health-enhancing physical activity
MET: Metabolic equivalent
NAPSE: National Action Plan for Sport and Exercise
NTFU: Netherlands Tour Cycling Union
OR: Odds ratio
SD: Standard deviation
SQUASH: Short QUestionnaire to ASsess Health-enhancing physical activity
T: Training session
TREND group: Transparent Reporting of Evaluations with Nonrandomized Designs group
