# Supplementary material for: The Start2Bike program is effective in increasing health-enhancing physical activity: a controlled study
Source: BMC Public Health. 2017 Jun 29;17:606. doi: 10.1186/s12889-017-4523-1 (PMC5493001; doi:10.1186/s12889-017-4523-1)
Supplement: Additional file 1: — Additional study results Start2Bike. In this file, some additional results of the Start2Bike study (not shown in the results section of this article) are presented. (DOC 27 kb) [file 12889_2017_4523_MOESM1_ESM.doc]

**Additional study results Start2Bike**

Table 1. Start2Bike group: cycling behavior and membership at the six-month measurement

|  | **Start2Bike group (n=79)** |
| --- | --- |
| Percentage of participants that is (still) mountain biking or road cycling | 75.9% |
| Percentage of participants that is a member of the cycling club that offered Start2Bike | 32.9% |
